# Seasonally increasing parasite load is associated with microbiota dysbiosis in wild bumblebees

**DOI:** 10.1128/msystems.01184-25

**Published:** 2025-11-18

**Authors:** Mark G. Young, Josefine Just, Ye Jin Lee, Thomas McMahon, James Gonzalez, Pilar Fuentes, Suegene Noh, David R. Angelini

**Affiliations:** 1 Department of Biology, Colby College 8439, Waterville, Maine, USA; China Agricultural University Education Foundation, Beijing, China

**Keywords:** microbiota, *Bombus*, bumblebee, *Crithidia*, dysbiosis

## Abstract

Gut microbiota often influence host defense against infection, but this relationship is incompletely understood in wild bumblebees. These critical pollinators host a characteristic core gut microbiota, yet field studies have offered conflicting insights into its association with *Crithidia bombi*, a prevalent trypanosomid parasite. To address this gap in our knowledge, we conducted an 3 year field survey, profiling the gut microbiota of 638 bumblebees from 9 sympatric species across diverse sites in Maine using 16S rRNA amplicon sequencing and qPCR for *C. bombi* detection. We confirmed a robust core bumblebee microbiota, identifying novel host-specific phylogenetic associations of bacterial amplicon sequence variants even among closely related host species. *C. bombi* infection was common and showed significant seasonal increases. We also found spatial variation, with higher prevalence in coastal regions. Crucially, increasing *C. bombi* infection load was consistently associated with microbiome dysbiosis. This dysbiosis was characterized by a depletion of core bumblebee-associated microbial taxa, notably *Apibacter* and *Gilliamella* (previously shown to be protective), and a corresponding increase in opportunistic, environmentally derived microbes like *Entomomonas*. While the core microbiota’s association with initial pathogen transmission appears minor, its depletion in severe infections strongly supports a correlation to host health in wild bumblebees.

**IMPORTANCE:**

The community of microorganisms in close association with an animal, its microbiota, can be important to its health. Understanding how microbiota composition relates to health and disease is an important goal with broad potential implications. Like most animals, bumblebees have a characteristic core gut microbiota. We have conducted a broad survey of bumblebees over 3 years to examine the interactions of microbiota composition with infection by an endemic trypanosomatid parasite. We found that the relative abundances of core microbes were inversely related to infection load, and that increased pathogen load was associated with the prevalence of novel microbes. These results are evidence of strong associations between bumblebees and their core microbiota and suggest a role in providing resistance to severe parasitism.

## INTRODUCTION

Because of their eusocial life history, bumblebees are an interesting case for investigation of the role of gut microbiota in disease susceptibility and defense. Eusocial corbiculate bees, including honey bees, stingless bees, and bumblebees, are primarily colonized by a small set of core bacterial genera, *Snodgrassella, Gilliamella, Bifidobacterium,* and *Lactobacillus* (Firm-4 and Firm-5) (1–3). Evolution of eusocial behavior was likely intertwined with the establishment of the corbiculate core microbiota, as the species of the core genera that colonize eusocial bees are phylogenetically distinct from taxa that colonize related non-eusocial bees (3, 4). Within colonies, social behavior is the main driver of the homogenization of microbiota communities. Newly eclosed bees must be raised alongside other adults, or their feces, to establish the characteristic microbiota (5, 6). Between colonies, foraging provides a route for the transmission of microbes (7). Pollinators deposit microbes on flowers (8), and gut-adapted bacteria are able to persist on flowers (9). Transmission between bumblebees and other pollinators is likely common, as bumblebees visit the same flowers within an area (10–12).

Parasitism is a focal point for bumblebee conservation and agricultural bee husbandry. Parasites do not pose a novel threat to bumblebees, and wild bees successfully mount immune responses against a diverse array of protozoa, nematodes, fungi, mites, prokaryotes, and viruses (13, 14). However, throughout the 20th century, numerous bumblebee species across Europe and North America have contracted in range, a sign of continuing decline in colony survival (15–19). Synergy with other stressors exacerbates parasite virulence, causing outsized impacts on host survival. This “context-dependent virulence” (20) is exemplified by a trypanosomatid endemic to Europe and North America, *Crithidia bombi*. Where *Crithidia* is endemic, forager infection rates can reach 80 to 100% by the end of summer (21–24). The parasite spreads within colonies through coprophagy (25, 26), and between colonies through foraging on the same flowers (27). Infections only become severe when hosts are exposed to concurrent stressors. A major source of stress for wild queen bees is hibernation, and infection by *C. bombi* reduces the likelihood of successful hibernation and subsequent colony rearing (20, 28). Context-dependent virulence can be replicated experimentally. While under standard feeding conditions infection does not affect mortality, it reduces survival by 50% under nutritional stress (29). Anthropogenic stressors, such as pesticide exposure or habitat loss, likely also drive context-dependent virulence, contributing to modern declines of bumblebee populations (30).

Organisms in the core bumblebee microbiota form a biofilm along the epithelium of the hindgut (31) and show genomic adaptation to the gut environment (6, 32–34). In the context of *Crithidia*, experimental results support a mechanistic role of the microbiota in resistance to severe infection. Workers raised under sterile conditions suffer far greater parasite loads than workers with normal microbial communities (5). Microbiota transplants between *Crithidia-*resistant and susceptible hosts have been shown to alter transcription of immune genes, with susceptible hosts adopting resistant-like gene expression after transplant (35). In *Bombus impatiens*, high relative abundances of the core taxa *Apibacter, Lactobacillus* Firm-5, and *Gilliamella* have been shown to inhibit severe infection (36).

Studies making use of wild bees indicate that these results may translate to wild bumblebee populations (36, 37). Diversity of non-core taxa has been associated with increased infection load, and the relative abundances of core taxa have been shown to have negative correlations with infection (5, 36, 37). However, the degree of inter-species and inter-site heterogeneity in the microbiota of wild populations remains unclear. Previous studies have disagreed on the significance of relationships between specific core taxa and infection load, some even finding no association between microbiota and infection status at all (38, 39). To address this issue, we have conducted a 3 year field survey, deeply sampling populations across coastal Maine and exploring how microbiota change with the landscape. The scale and breadth of our data set have enabled us to examine trends of microbiota composition with *Crithidia* infection, as well as isolate inter-season, inter-site, and inter-species variation. We found consistent seasonal changes in bumblebee microbiota composition and identified variation in the relative abundances of core taxa among host species. Controlling for external sources of variation, parasite load had an inverse relationship with the relative abundances of a few core taxa, and infection was characterized by an increase of what appeared to be bacterial opportunists.

## RESULTS

### Wild bumblebee species share a core microbiota community

**Fig 1:**
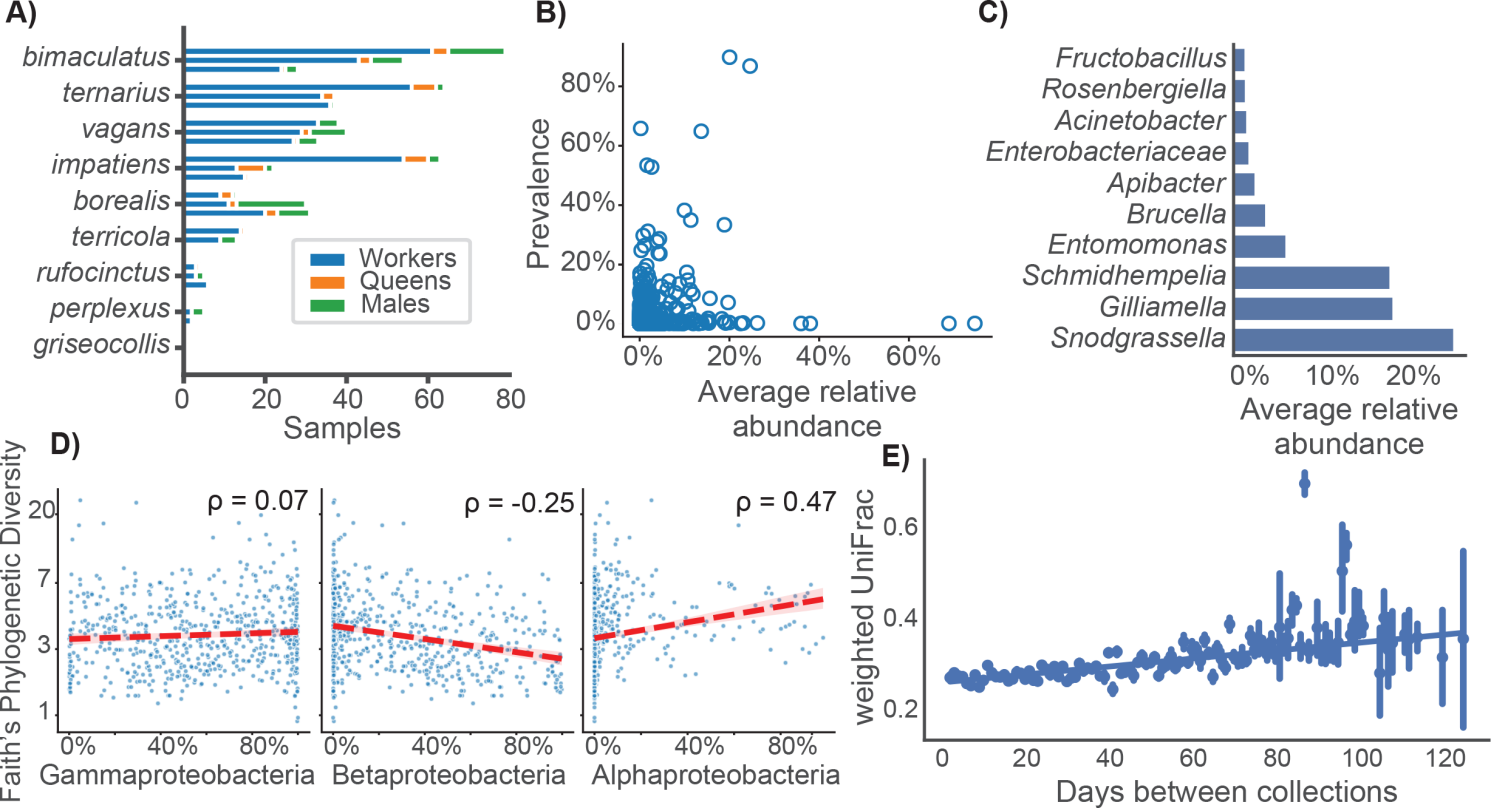
Wild bumblebees share a genus-specific microbiota community. (**A**) Wild species and castes are unequally represented in the field survey data set. The prevalence of species is represented by horizontal bars, broken down by caste (color) and year (rows, descending 2017–2019). (**B**) Amplicon sequence variant prevalence across samples vs average non-zero relative abundance. (**C**) Average relative abundance of the most frequently identified microbial genera across all samples. (**D**) Relative abundance of the three most abundant classes vs Faith’s phylogenetic diversity. Spearman correlations are shown in the top right corner of each plot. (**E**) The difference in microbiota composition between samples was dependent on the difference in their dates of collection (Spearman’s ρ = 0.10, Mantel test, *P* < 0.001). The difference in collection date was calculated from day-of-year dates, in order to test for trends consistent across each year of the field survey.

We used 16S ribosomal RNA amplicon sequencing to profile the gut microbiota communities of 638 bumblebees collected during the course of a 3 year field survey in Maine. Our sample set included workers (*n* = 505), queens (*n* = 45), and males (*n* = 88) of nine sympatric bumblebee species from 64 ecologically diverse sites (Fig. 1A). We targeted the V6–V8 region of 16S for taxonomic identification. Sequencing samples had a mean depth of 51,927 paired-end reads after quality control.

Consistent with prior studies, a small number of microbial taxa accounted for nearly all of the bumblebee microbiota. Of 3,331 amplicon sequence variants (ASVs) detected within our sample set, just 172 were observed in multiple samples at an average relative abundance greater than 1% (Fig. 1B; Table S1). Samples were overwhelmingly colonized by ASVs classified as Gammaproteobacteria or Betaproteobacteria (mean 56%, 28% relative abundance). Identified ASVs included a small number of genera previously annotated as being part of the bumblebee core microbiota (Table S2), three of which (*Snodgrassella, Schmidhempelia,* and *Gilliamella*) accounted for 66% of relative abundance on average (Fig. 1C). A reduction in Betaproteobacteria was associated with an increase in α-diversity (Spearman’s ρ = −0.25), as was an increase in Alphaproteobacteria (Spearman’s ρ = 0.45; Fig. 1D).

We tested for stratification of microbiota structure by host species, host caste, collection site, and collection month with marginal PERMANOVA. All factors explained significant variation in the weighted UniFrac dissimilarity between samples, after multiple hypothesis correction (*P* < 0.05; Table 1). However, the size of the effects was small, and samples did not show obvious clustering on principal coordinate plots (Fig. S1). We used Mantel tests to test the monotonicity of the significant relationships. Difference in collection date (year-free) was a significant predictor of β-diversity (Spearman’s ρ = 0.10, *P* < 0.001; Fig. 1E; Table 2), indicating bumblebee gut microbiota changed consistently across each summer. However, phylogenetic dissimilarity (sum of branch lengths) and the distance between collection sites did not show monotonic relationships with β-diversity.

**TABLE 1:** Weighted UniFrac distance marginal PERMANOVA (adonis2)

	df	SS	*R* ^2^	F	*P*	Bonferroni-adjusted *P*
Species	8	1.998	0.06201	5.9219	0.001	0.004
Site × year	87	5.611	0.17413	1.5292	0.001	0.004
Month	4	0.446	0.01383	2.6412	0.001	0.004
Caste	2	0.22	0.00683	2.6083	0.006	0.024
Residual	521	21.975	0.68192			
Total	622	32.225	1			

**TABLE 2:** Weighted UniFrac Mantel correlation

	Correlation	*P*-value	Samples
Phylogeny	−0.01490552887	0.508	638
Geography	−0.002005136825	0.914	563
Day	0.1012259204	0.001	638

### The phylogenetic divergence of bee-associated ASVs correlates with those of their hosts

We next looked for evidence of phylogenetic associations between members of the bumblebee microbiota and their hosts. We used weighted UniFrac to quantify the differences in ASV populations between samples and tested for correlations with host phylogeny using Mantel tests. Such associations have been identified in larger-scale comparisons between honeybees, bumblebees, and stingless bees, with more closely related bees hosting more closely related strains (3). We identified a set of 31 moderately prevalent bacterial genera (detected in >10% of samples) in our bumblebee-specific data set. Individual samples were generally colonized by just one or two ASVs of the same genus, rather than the full diversity of observed ASVs (Table S3). After controlling for inter-site variation, we found significant correlations for 10 genera in which more closely related bumblebees hosted more closely related ASVs (Mantel test stratified by collection site, *P* < 0.05; Fig. S2; Table S3). These included ASVs of *Gilliamella* and *Schmidhempelia*. Both taxa are considered members of the bumblebee core microbiota and were detected in >90% of samples. These correlations indicate phylogenetic diversification of endosymbionts alongside their bumblebee hosts, and that for these 10 genera, host specificity had a greater effect on microbiota composition than local transmission.

### *Crithidia* infections are common throughout Maine and increase in prevalence over the course of the summer

**Fig 2:**
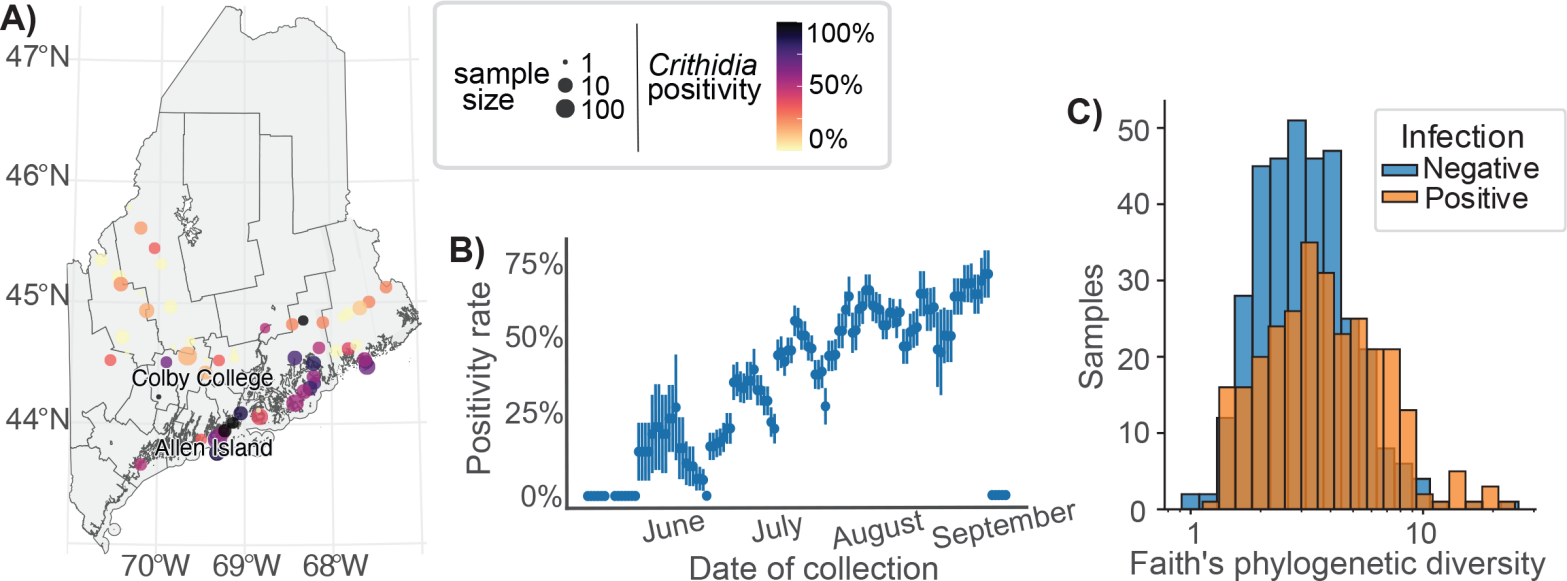
*Crithidia* infection was common and seasonal. (**A**) Map of Maine with markers for collection sites, sized by number of samples collected per site, and colored by *Crithidia* infection prevalence. (**B**) *Crithidia* infection rates increased over the course of the summer. Positivity rates were estimated as the proportion of positive samples collected within 5 day bins. Rate estimates include samples from all 3 years of the field survey. Standard deviations of estimates are shown as vertical bars. (**C**) *Crithidia*-positive samples had greater Faith’s phylogenetic diversity (Mann-Whitney U-test, *P* < 10^−5^).

Bumblebees from all three summers of the field survey were infected with the parasite *Crithidia bombi*. We classified infections with qPCR, using 1,000 copies/µL as a standard for detection. Samples classified as positive varied more than 10,000-fold in concentration-normalized *C. bombi* load (copies/ng; Table S7). The overall *C. bombi* infection positivity rate was 45% but varied significantly between local populations. We selected the five sites that were most deeply sampled (≥15 samples/summer) for a comparison of infection rate between local populations: two geographically isolated offshore islands (Vinalhaven and Allen Island), two islands reachable by bridge (Great Wass and Swans Island), and one mainland site (Colby College). The single-summer positivity rates were significantly different between sites (ꭓ^2^ test, *P* < 2.7 × 10^−12^) and greatest for the island sites (Fig. 2A). Over 50% of samples collected from Allen Island, Great Wass Island, and Swans Island tested positive, while only 15.6% from Colby College were positive for *Crithidia*.

Generally, infection rates were greatest in coastal areas and increased over the course of each summer (Fig. 2A and B). We used a generalized linear mixed-effects model to measure the dependence of infection status on collection date, latitude and longitude of collection site, and caste, with random effects to capture inter-site and inter-species variation (Table S4). To capture inter-species differences, we used independent and identically distributed random intercepts (i.i.d.), as well as a correlation structure proportional to the phylogenetic dissimilarity between hosts. The phylogenetic encoding explained roughly one-fifth of the total variation explained by the site term (*P* = 0.025), while the contribution of the i.i.d. encoding was negligible (Table S4). There was no difference in infection positivity rate between castes, but the odds of infection increased significantly at more southern and eastern sites, and by 2.2% (*P* < 0.001) for each day of the summer. Even after controlling for trends with latitude and longitude, the random effect of site explained a large and significant proportion of total variation (likelihood ratio test; *P* < 1 × 10^−4^). In contrast, the variation explained by host species was relatively small.

### *Crithidia* infections were associated with changes to the relative abundances of a small number of microbial taxa

**Fig 3:**
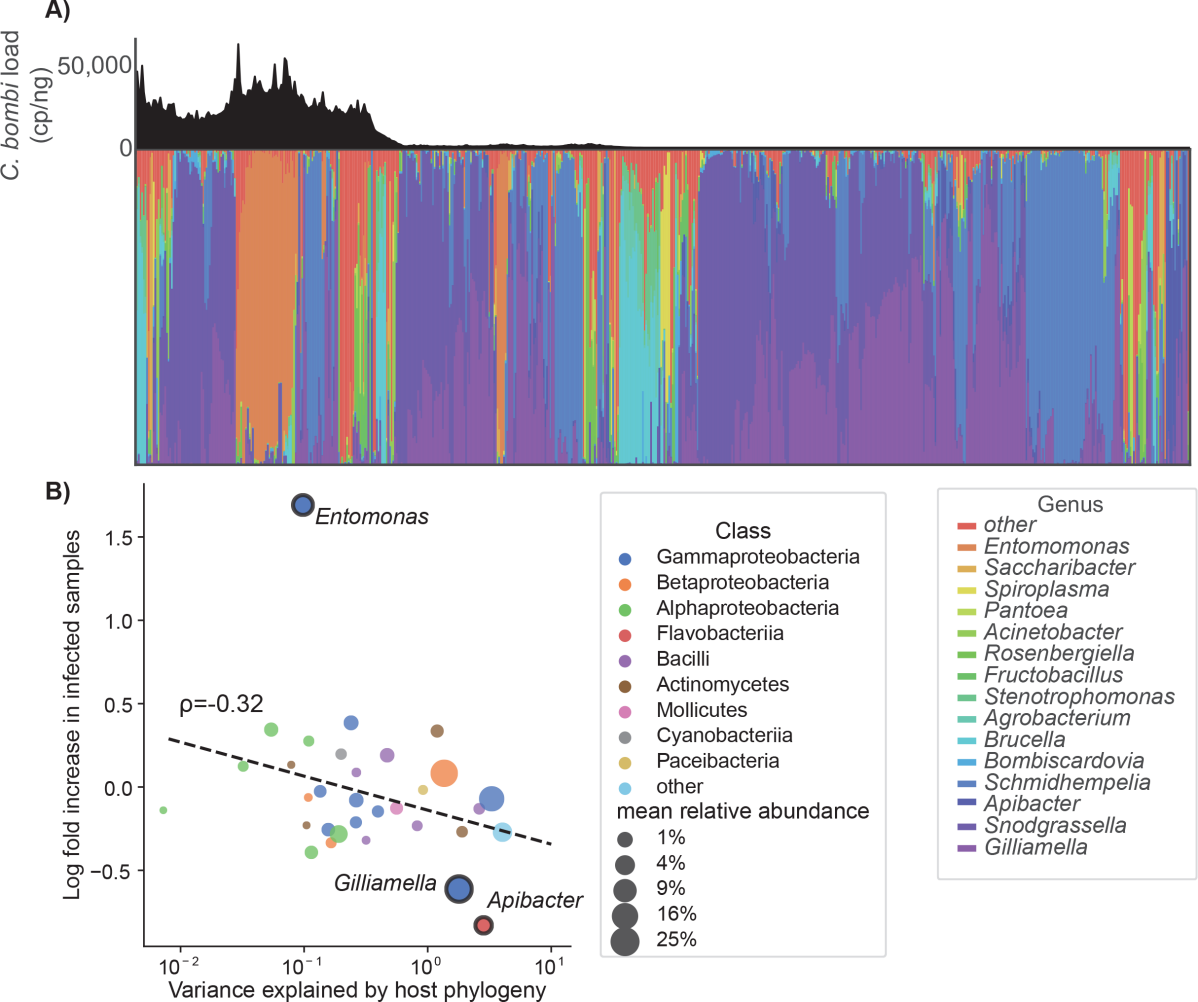
*Crithidia* infection was associated with gut microbiota. (**A**) The relative abundances of the 15 most abundant microbial taxa are shown for all 638 samples. Samples are grouped by *Crithidia* load (non-infected, below median load, above median load) and sorted within groups by agglomerative clustering on weighted UniFrac distance. Average *Crithidia* load was estimated using a sliding window with a radius of 20 samples and is shown above the taxonomy plot. (**B**) Linear mixed-effects models were used to assess the association between the relative abundances of individual taxa with *Crithidia* infection, collection date, collection site, and host species. Points represent the variance in relative abundance explained by phylogenetic random effects (*x*-axis) and the estimated log-fold change between negative and positive samples (*y*-axis). Significantly differentially abundant taxa (*P* < 0.1) are labeled. Trendline and correlation coefficient are plotted.

The relative abundances of a small number of microbial taxa were different between *Crithidia-*infected and non-infected bees, suggestive of infection-associated dysbiosis (Fig. 3A). We tested whether *Crithidia* infection was associated with the 31 genus-level and 10 class-level taxonomic bins found across 10% or more samples, and an “other” bin, containing (i) less prevalent taxa and (ii) ASVs that could not be fully assigned. The genus-level “other” bin contained 479 genus-assigned taxa and 150 higher-level bins, representative of 6.0% of average relative abundance, and the class-level bin contained 61 low-prevalence classes and six higher-level bins, for 0.14% of average relative abundance. To test for differential abundance of these taxonomic bins, we used linear mixed-effects models, as described in MaAsLin (40), with fixed and random effects to control for the effects of host species, collection date, and collection site.

Across all genera included in modeling, the degree of association with host phylogeny had a negative correlation with association with infection (ρ = −0.32, *P* = 0.07; Fig. 3B), indicating that non-infected bees host a more species-specific microbiota composition, while infected bees are more likely to host more random taxa. Infected samples also had greater Faith’s phylogenetic diversity (Fig. 2C; Mann-Whitney U-test, *P* < 10^−5^), further indicating the presence of additional non-core taxa.

Among specific microbial taxa that changed in relative abundance with infection, the genus *Entomomonas* (family Pseudomonadaceae) and the class-level grouping of “other” taxa were positively associated with infection (Benjamini-Hochberg [BH] correction, *P* < 0.001; Table S5). The genera *Apibacter* and *Gilliamella* were negatively associated with infection, though to a lesser degree (*P* < 0.10). Qualitatively, the two health-associated genera appeared to be consistent and prevalent members of the bumblebee microbiota, while infection-associated *Entomomonas* and class-other were only found at high relative abundance in small clusters of primarily *Crithidia*-positive samples (Fig. 3A).

Many microbes also changed in relative abundance over the course of the summer, consistent with the shift in weighted UniFrac diversity noted above. These included the infection-associated genus *Entomomonas* (increase), mirroring the date-dependent rise in *Crithidia* infections (*P* < 0.05, BH correction; Table S5; Fig. S3). However, across the full set of taxa, there was no collinearity between *Crithidia* and collection date associations. The health-associated *Gilliamella* and *Apibacter* also increased in relative abundances at later collection dates. In total, 22 genera (51% relative abundance) and 3 classes (59% relative abundance) were significantly associated with collection date. Among them, there was no correlation between date and infection association (Spearman’s ρ = 0.21, *P* = 0.31).

### Severe infection is associated with increasingly dysbiotic microbiota composition

**Fig 4:**
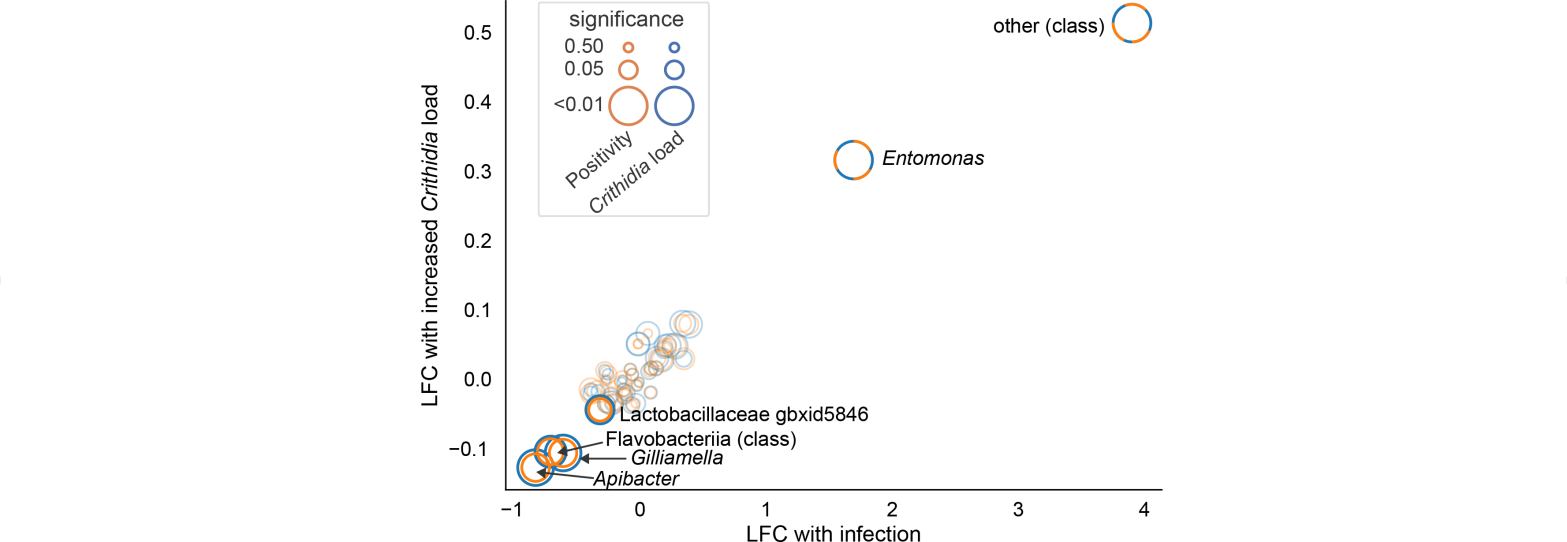
Microbial taxa associated with infection were also associated with increased infection load. Points indicate the differential abundance of individual microbial taxa between non-infected and infected samples (*x*-axis) and with increased infection load among *Crithidia*-positive samples (*y*-axis). Points are sized by significance, with the blue circle indicating the significance of the relationship with infection load and the orange circle indicating positivity. Microbial taxa for which either relationship was significant (*P* < 0.1) are bolded and labeled. Class- and genus-level taxonomic groupings were used for differential abundance testing.

Among *Crithidia*-positive samples, there was great heterogeneity in infection load. Positive samples exhibited a more than 10,000-fold range in infection load (Table S7). We used linear mixed-effects models to assess the relationship between infection load as a continuous dependent variable and the relative abundances of microbial taxa, using only samples that passed the positivity threshold. As in the earlier model, we controlled for host species, collection date, and collection site. Infection load was associated with increases in the relative abundance of *Entomomonas* and class-other, and decreases in *Gilliamella, Apibacter,* and class Flavobacteria (*P* < 0.05, BH correction; Fig. 4; Table S6). Across the full set of taxa, there was a strong correlation between the fitted values from both sets of models (Spearman’s ρ = 0.80, *P* < 10^−9^), indicating that changes in relative microbial taxa abundances with increased severity largely mirrored the differences between non-infected and infected bees.

## DISCUSSION

### Evidence for a core bumblebee microbiota

The results of our Maine bumblebee field survey were consistent with prior findings of a robust and coevolved bumblebee gut microbiota. Samples from all 9 *Bombus* species were primarily colonized by bacterial genera previously annotated as being part of the core bumblebee microbiota, mostly of the classes Gammaproteobacteria and Betaproteobacteria (Table S2; Fig. 1C). The resolution of our study was limited by our use of short-read 16S amplicons and the microbial taxa represented in the GreenGenes (v.2022.10) and BEExact (v.2023.01.30) databases used for taxonomic classification. We were unable to differentiate between phylotypes of the genus *Lactobacillus* and identify the genus *Parasaccharibacter*, both considered low-abundance members of the core microbiota (3). In spite of these limitations, we still retained a high degree of taxonomic sensitivity. Across the full data set, we identified 3,331 unique ASVs, which corresponded to 510 fully classified, genus-level taxonomic bins. *Snodgrassella* and *Gilliamella* were the most frequently observed microbes, colonizing just over 98% and 96% of samples, respectively (Table S2). Similarly high prevalences of these core taxa have been observed in other surveys of field-caught bees (36, 37, 41).

The field survey data set additionally provided evidence for microbiota divergence between sympatric bumblebee species. Prior characterizations of the bumblebee microbiota have focused on the genus as a whole, often contrasted against the other tribes of corbiculate bees. We found that members of the “bumblebee core microbiota” (including *Snodgrassella, Gilliamella,* and *Schmidhempelia*) varied in relative abundance between sympatric bumblebee species. For these core taxa, divergence was proportional to phylogenetic dissimilarity between hosts (Table S5; Fig. 3B). At a more granular level, we also found evidence of host specificity of ASVs of 10 prevalent genera (found in >10% of samples), including bee-associated *Gilliamella, Bombiscardovia,* and *Bifidobacterium* (Fig. S2; Table S3). Similar phylogenetic associations have been reported in large-scale comparisons of corbiculate bees (3), and confirmed experimentally, with strains isolated from bumblebees and honeybees (31). To our knowledge, this is the first observation of evidence for host specificity within bumblebees. Notably, we did not detect a relationship between *Snodgrassella* ASV and host phylogeny (Table S3). There was relatively little ASV diversity within *Snodgrassella*, with one major ASV accounting for ~80% of all *Snodgrassella*-relative abundance (Table S1). Possibly, this could reflect a below-average mutation rate at the 16S loci chosen for amplification (V6–V8).

### Gut flora change with the seasons

Seasonal variation in bumblebee ecology was reflected in the microbiota. The bumblebee life cycle can be divided into two major phases: colony formation in the summer and hibernation in the winter. Only the gynes (reproductive females) hibernate before emerging in the spring to form colonies of their own. Over the winter, core taxa decrease in relative abundance, while the microbiota increases in richness and evenness (42, 43). In the summer phase of the life cycle, the bumblebee microbiota undergoes selective pressure from diet and is homogenized between colonies through pollination-mediated exchange of microbes. Consistent with local exchange, we found that the collection site of samples was a significant determinant of their microbiota communities (Table 1). We also found date-dependent variation in community structure that was consistent across all 3 years of the field survey, implicating annual life-cycle trends and floral rewards as potential driving factors (Fig. 1E; Table 2). Further supporting the role of host ecology in determination of the microbiota, we found significant inter-caste variation in composition (Table 1), after controlling for confounding effects of collection date.

### *Crithidia* infection is associated with dysbiosis of the gut microbiota

*Crithidia* infections were seasonal and highly prevalent within our data set. In contrast to previous reports of mid-summer occurrences of peak infection (44), we found infection rates increased until collections ended in early September. Positivity rate climbed linearly from around 10% in May to near 70% in September (Fig. 2B), consistent with continual parasite transmission within and between local colonies. Since we did not account for the age of individuals, age-related exposure or immune differences may also potentially contribute to this trend. Infection positivity rates were also highly variable between sites, possibly as a result of variable local colonization by the parasite or population bottlenecks caused by overwintering. Coastal sites had far greater *Crithidia* loads on average. Latitude and longitude were significant predictors of forager positivity rates, even after controlling for other sources of variation (Table S4; Fig. 2A). In other work, *Crithidia* prevalence has been associated with urban density (45). Urban environments often have fewer flowers, driving increases in forager density and opportunity for pathogen transmission. Coastal Maine is more densely populated by people than the interior, which may be driving the observed relationship. Alternatively, the relationship could also be reflective of the milder winters of coastal Maine in comparison to the interior. Infected gynes overwinter with the parasite, and if they survive, spread the infection in the spring (20). If colder temperatures induce stress on infected gynes, increased overwinter mortality would be expected (20), and decreased survival of infected gynes could be a mechanism by which *C. bombi* prevalence is reduced in interior Maine. Interestingly, this spatial variation was not reflected in the broader microbiota. Despite high inter-site variation in community composition, there was no monotonic trend with the distance between collection sites, indicating that broader trends in climate or urbanization did not have a consistent influence on the microbiota (Table 2).

Differences in microbiota community between infected and non-infected bees were indicative of a *Crithidia*-associated dysbiosis that worsened with increasing infection load. We found that infection positivity was associated with increases to community α-diversity and the fraction of the microbiota composed of diverse, low-abundance “other” taxa (Fig. 2C and 3A), consistent with prior findings of associations between the non-core microbiota and *Crithidia* (36, 37, 46). *Entomomonas*, the *Crithidia*-associated genus identified through linear modeling, was inconsistently found in infected samples (Fig. 3A) and strongly associated with specific collection sites (Table S5), indicating that it may just be a common, environmentally derived opportunist able to colonize the dysbiotic bee microbiota.

Increasingly severe infections were characterized by further outgrowth of non-core taxa, accompanied by a depletion of bumblebee-associated core genera (Fig. 4). From this data set alone, it is unclear whether these changes to the microbiota were a cause or an effect of *Crithidia* infection. However, two of the genera identified as negatively associated with infection load, *Apibacter* and *Gilliamella*, were found to be protective against infection in prior experimental work (36), supporting their active roles in resistance to severe infection in wild bumblebee populations. The core microbiota may have a relatively minor relationship with initial pathogen transmission; however, changes to its composition were primarily associated with severe infections.

### Conclusion

Here, we report the results of a 3 year field survey of the gut microbiota dynamics of wild bumblebees, in the context of infection by an endemic trypanosomatid, *Crithidia bombi*. We found that the relative abundances of core taxa were inversely related to infection load, and that non-core microbial taxa increased in relative abundance in infected samples. Additionally, we found evidence for diversification in the microbiota between sympatric bumblebee species and patterns of seasonal variation in community structure that were consistent across all 3 years of the field survey. These results are evidence of strong associations between bumblebees and their endosymbionts, and of the contribution of the core microbiota to resistance to severe parasitism.

## MATERIALS AND METHODS

### Sample collection

Foraging bumblebees were collected throughout the state of Maine during summer 2017, 2018, and 2019 (Table S7). We did not visit all sites evenly; some were longitudinally sampled during each year of the field study, while others were visited opportunistically. Individuals were photographed at the time of capture and stored at −80°C before dissection to remove the gut. Species and caste were confirmed with reference to Williams et al. (47). Maxwell 16 DNA purification kits (Promega, Madison, WI) were used for DNA extraction from gut samples. DNA extractions were performed separately after each field season. Extracted DNA was stored at −20°C prior to sequencing.

### Screening and quantification of *Crithidia bombi*

We screened bumblebee gut DNA samples for *Crithidia bombi* infections with quantitative PCR on a CFX96 Touch real-time thermocycler using iTaq Universal SYBR Green Supermix (Bio-Rad, Hercules, CA) and primers for *C. bombi GAPDH* (Cb´gapdh-52F GCGTACCAGATGAAGTTTGATACG; Cb´gapdh-147R AAGCACATCCGGCTTCTTCA). We used 1,000 copies/µL DNA as a threshold for positivity. Samples were initially screened in batches in order to reduce the total number of reactions. We used a batch size of four and ran batches in duplicate. We then individually screened samples from positive batches in triplicate. We used linear regression models fit to qPCR standards to estimate infection load of individual samples in copies per microliter. We converted copies per microliter to copies per nanogram by scaling by the DNA concentration of samples used for qPCR (ng/µL).

### Microbiota sequencing

DNA samples were shipped on ice packs to the Centre for Comparative Genomics and Evolutionary Bioinformatics at Dalhousie University (Halifax, Canada). Samples were used to prepare paired-end 2 × 300 bp MiSeq libraries, with PCR amplification targeting variable regions 6–8 of the bacterial 16S ribosomal RNA gene (48). Of 732 bumblebees collected, 638 samples passed standard benchmarks for library quality and were included in subsequent analyses (Table S6).

### ASV processing and taxonomic classification

We processed the demultiplexed amplicon sequencing data with QIIME2 (v.2024.10). We trimmed the first 34 bp from each read and truncated forward and reverse mates to 280 and 240 bp, respectively, before forming ASVs and removing chimeras with DADA2 (v.1.1.0) (49). We then classified ASVs with a naive Bayes classifier trained on the BEExact database (v.2023.01.30) of full-length sequences, using a minimum posterior probability of 0.7. For ASVs that could not be classified to the genus level with the BEExact classifier, we performed a second round of classification with the GreenGenes database of full-length 16S sequences (v.2022.10), using the GreenGenes classifications when they agreed with and extended the BEExact classifications. There were no cases where the GreenGenes and BEExact classifiers disagreed on higher-level classifications (e.g., differing family classifications), only cases where one classifier provided greater specificity. In cases where clades had been renamed or moved, we used the BEExact nomenclature.

### Diversity calculation

We used phylogeny-aware weighted UniFrac distances for β-diversity analyses. To build a *de novo* phylogeny, we created a multiple sequence alignment of all ASVs with MAFFT (v.7.505) (50) and masked positions with conservation less than 40%. We then created an unrooted tree with FastTree2 (51) and midpoint-rooted this tree. UniFrac distance calculations were implemented in QIIME2. We calculated Faith’s phylogenetic diversity, also using the rooted tree and a feature table down-sampled to a depth of 4,000 ASVs, which included 631 (98.9%) of 638 samples.

### Host metadata

We measured the dependence of microbiota composition on host species, collection site, and collection date. To quantify the phylogenetic dissimilarity between bumblebee species, we used the sum of branch lengths from the bumblebee phylogeny reported in Cameron and Hines (52). To quantify the difference in collection site between samples, we used the distance in kilometers between individual bumblebee collection sites and also categorically encoded collection sites. Categorical sites had maximum radii of 5 km, which has been reported to be the maximum foraging range of worker bees (13). We used day-of-year encoding for collection dates, as well as categorical offsets for each year of the field survey.

### Statistical analysis

All statistical analyses were performed in R v.4.1.3 (53). We used linear models to assess variation in *Crithidia* infection rate and to test for differential abundance of microbial taxa. For both applications, we fit models with the pglmm function from the R package phyr (54). We used pglmm for its support of generalized linear mixed-effects modeling with phylogenetic random effects. For modeling variation in *Crithidia* infection rates, we fit the following model with a binomial link function:


Crithdia∼ days_Since_May1+caste+(1|species__)+(1|site_year)

The variables were defined as follows.

*Crithidia:* Infection status (positive/negative) of individual samples
*days_Since_May*1: The year-independent collection date of samples, measured as the days between sample collection and May 1st of the same year.
*caste:* sample caste (male/worker/queen)
(1*|species__*): The phylogenetic effect, consisting of independent and identically distributed intercepts for bumblebee species, as well as a covariance structure proportional to the phylogenetic dissimilarity between species (sum of branch lengths).
(1*|site_year*): Independent and identically distributed random intercepts for collection sites. Collection sites were encoded separately for each year (e.g., AllenIsland_2017, AllenIsland_2018, AllenIsland_2019), in order to capture year-specific variation.

Prior to log-transformation of relative abundances, we additively smoothed zero values with a pseudocount equal to half the smallest non-zero relative abundance. We used Benjamini-Hochberg false discovery rate-corrected *P*-values to assess significance of associations. We corrected for false discovery rate separately for the genus- and class-level models. For all modeling applications, we used likelihood ratio tests (implemented in phyr) to quantify the significance of the variation explained by random effects. In the discussion of differential abundance testing results, by “species random effect,” we refer to the covariance structure proportional to phylogenetic dissimilarities between host species. All figures were created with Python v.3.8.16 using seaborn v.0.12.2 and matplotlib v.3.6.0 libraries.

## Data Availability

Code and data involved in this analysis are available online at https://github.com/aphanotus/bombus.landscape.

## SUPPLEMENTAL MATERIAL

Supplemental Figures msystems.01184-25-s0001.pdf Figures S1 to S3.

Supplemental Tables msystems.01184-25-s0002.xlsx Tables S1 to S7.

ASM does not own the copyrights to Supplemental Material that may be linked to, or accessed through, an article. The authors have granted ASM a non-exclusive, world-wide license to publish the Supplemental Material files. Please contact the corresponding author directly for reuse.
