# Supplementary material for: Seasonally increasing parasite load is associated with microbiota dysbiosis in wild bumblebees
Source: mSystems. 2025 Nov 18;10(12):e01184-25. doi: 10.1128/msystems.01184-25 (PMC12710312; doi:10.1128/msystems.01184-25)
Supplement: Supplemental Figures — Figures S1 to S3. [file msystems.01184-25-s0001.pdf]

Supplementary Figures

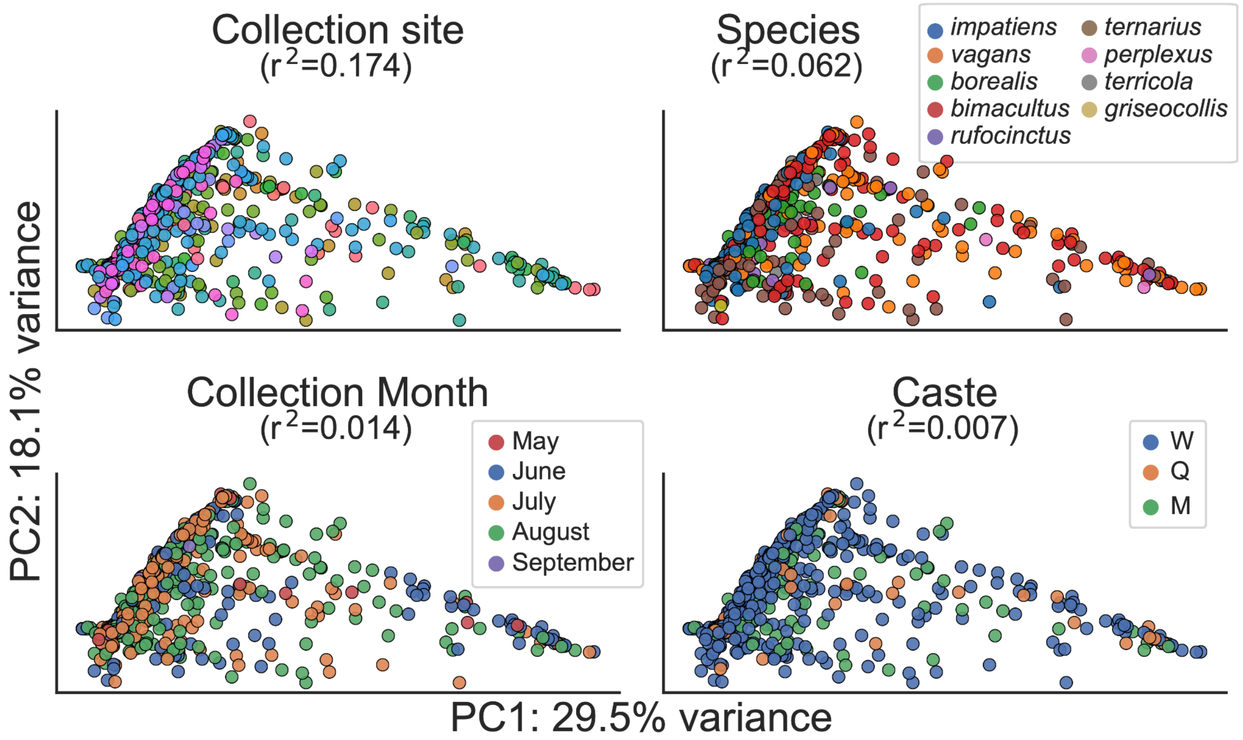

Supplementary Figure 1: Weighted UniFrac PcoA. Collection site, host species, collection month, and host caste were all significant determinants of bumblebee microbiota composition (PERMANOVA, $p < 0.05$). However, the size of the effects were small, and samples did not visibly cluster. Points on PcoA plots are colored by categorical metadata. The legend for the collection site PcoA is withheld due to size (90 sites).

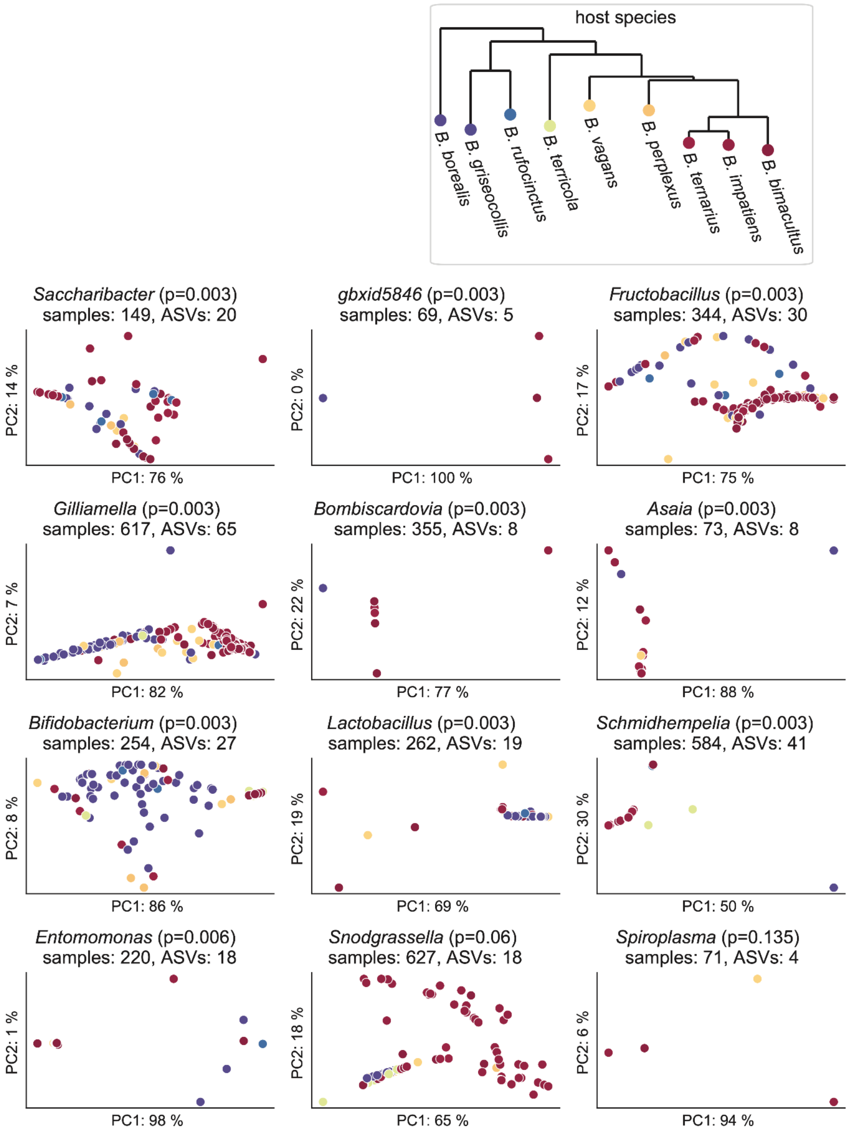

Supplementary Figure 2: ASV dissimilarity was associated with host divergence for ten genera . Subplots show the first two principal components of the weighted UniFrac dissimilarity between genera-specific fractions of samples. Samples are colored according to host species, using the phylogeny reported in Cameron and Hines (2007). Significance of association between weighted UniFrac divergence and host phylogeny was assessed with Mantel tests for correlation with stratification by collection site and year. False discovery rate corrected p-values are shown in subplot titles, as well as genus prevalence in the dataset and the number of unique ASVs assigned to the genus.

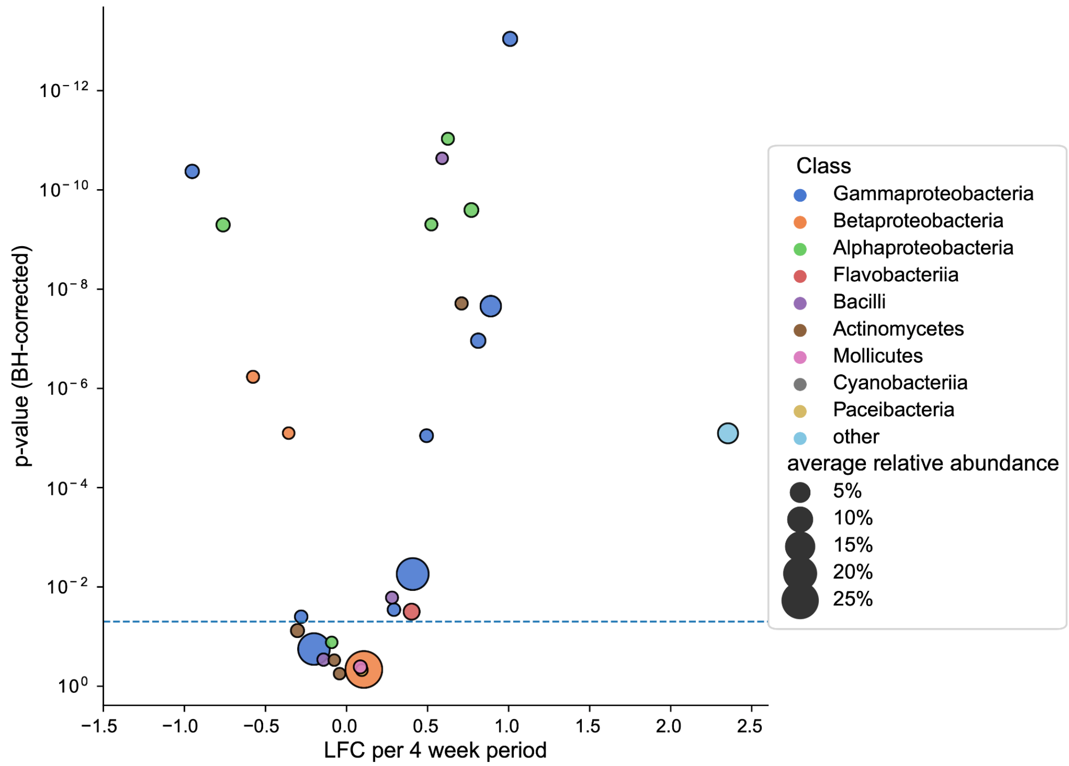

Supplementary Figure 3: Relative abundances of genera were associated with collection date. Points represent specific genera, colored by class and sized by average relative abundance. The chosen significance threshold ($\alpha = 0.05$) is indicated by a dashed horizontal bar.
